# Biportal endoscopic spine surgery for treatment of thoracolumbar intervertebral disc herniation in 13 dogs

**DOI:** 10.3389/fvets.2025.1638065

**Published:** 2025-07-02

**Authors:** Yongsun Kim, Ji-Hey Lim, Yebin Ryu, Dae Jung Choi

**Affiliations:** ^1^Department of Veterinary Surgery, BON Animal Medical Center, Suwon, Republic of Korea; ^2^Department of Surgical and Radiological Sciences, University of California Davis, Davis, CA, United States; ^3^Himnaera Hospital, Busan, Republic of Korea

**Keywords:** biportal endoscopic spine surgery, intervertebral disc herniation, BESS, IVDH, dog

## Abstract

**Objective:**

This study aimed to describe the technique and evaluate the clinical outcomes of utilizing a biportal endoscopic spine surgery (BESS) for the treatment of thoracolumbar intervertebral disc herniation (IVDH) in dogs.

**Methods:**

Thirteen client-owned dogs diagnosed with single level thoracolumbar IVDH using magnetic resonance imaging were included. A mini-hemilaminectomy was performed using the BESS system. Briefly, the dogs were positioned in sternal recumbency and two portal skin entry points were confirmed under fluoroscopic guidance. The endoscopic portal provided continuous irrigation and visualization, while the instrumental portal allowed for instrument manipulation and disc removal. Pre- and postoperative neurologic status, operation time, perioperative complications were recorded and analyzed.

**Results:**

The dogs ranged in age from 4 to 11 years (median 7.5 years) and weighed ranging from 5.0 to 9.1 kg (median 7.4 kg). Clinical presentations ranged from ambulatory paraparesis to non-ambulatory paraparesis. The BESS approach enabled effective spinal cord decompression and removal of extruded disc material without intraoperative complications. No cases required conversion to open hemilaminectomy. The average operation time was 53 ± 10.5 min. At 6 weeks postoperatively, all dogs exhibited normal neurological function, and no complications were reported.

**Conclusion:**

These clinical findings support that minimally invasive BESS is a safe and feasible technique for treating thoracolumbar IVDH in small-breed dogs. The BESS approach offers an effective surgical alternative for the treatment of thoracolumbar IVDH in canine patients.

## 1 Introduction

Thoracolumbar intervertebral disc herniation (IVDH) is a common causes of acute spinal cord compression in dogs, particularly in chondrodystrophic breeds (1). Affected dogs often present with varying degrees of neurological deficits, ranging from back pain and ataxia to complete paralysis. The current standard of care for severe thoracolumbar IVDH is surgical decompression, most commonly via a hemilaminectomy (2, 3). Although traditional open hemilaminectomy is generally effective in relieving spinal cord compression and can result in favorable neurological recovery, it is an invasive procedure. The open approach requires a relatively large skin incision, substantial paraspinal muscle dissection, and removal of vertebral bone, all of which can contribute to postoperative pain, prolonged recovery, and increased risk of complications such as hemorrhage, instability, or infection (4).

Minimally invasive spine surgery techniques have been widely adopted in human medicine for the treatment of disc herniations and spinal stenosis. These approaches offer several advantages, including reduced tissue trauma, smaller incisions, reduced postoperative pain, and faster return to function (5, 6). Biportal endoscopic spine surgery (BESS) is a relatively new technique that utilizes two small portals to perform of spinal canal decompression. Clinical studies in human have shown that BESS can achieve outcomes comparable to those of open surgery for lumbar disc herniation and spinal stenosis, while also minimizing paraspinal muscle damage and reducing the size of surgical wounds (7, 8). Additionally, a unique advantage of the biportal technique compared to other minimally invasive methods is its familiarity to surgeons, allowing for broader application in clinical practice (9).

In veterinary medicine, minimally invasive approaches to the spine surgery have been introduced but investigations remain limited until recently (10). A mini-open approach using a surgical microscope or endoscope-assisted hemilaminectomy have been described in both cadavers and live animals (4, 11–14). The use of full endoscopic approach for thoracolumbar decompression in dogs been reported only a few studies, primarily in cadaveric models and small case series (15–17). However, to date, the application of BESS in dogs has not been documented in the veterinary literature.

Therefore, this report aims to describe the surgical technique of BESS for treating thoracolumbar IVDH in dogs and to present clinical outcomes from a series of cases treated with this novel minimally invasive procedure. We hypothesized that the BESS technique could be adapted for use in dogs to achieve adequate decompression of the spinal cord in cases of thoracolumbar disc extrusion.

## 2 Materials and methods

### 2.1 Case selection

Dogs diagnosed with a single-level thoracolumbar IVDH confirmed by magnetic resonance imaging (MRI) were included for BESS procedure between September 2023 and January 2025. Informed consent was obtained from the owners of all enrolled dogs. Dogs with multi-level disc herniation or MRI evidence suggestive of myelomalacia were excluded from the study. Owners were informed of the potential benefits and risks associated with BESS, including the limited clinical data available on this innovative procedure in dogs. Collected data included each dog’s signalment, medical history, and presenting neurological signs. Surgical reports and patient records were reviewed to gather information on the surgical techniques employed, as well as intraoperative and postoperative complications. Follow-up data were obtained during recheck appointments to assess patient progress.

### 2.2 Surgical instruments

The following surgical equipment were used to perform BESS for thoracolumbar decompression. Creation of the endoscope and working portals involved the use of an 18 G spinal needle and 0.8 mm K-wire to localize the operation site, followed by a 5-mm dilator to assess the docking point. The endoscopic system consisted of a 4-mm diameter, 140 mm length, 0-degree rigid endoscope (Stryker, Portage, Michigan) equipped with irrigation sheath. A 5-mm cannula was used to establish the working port. Additional instrumentation included 1-mm tip rotating Kerrison Rongeurs, 1- or 2- mm blunt-angled nerve root retractor, and a radiofrequency (RF) system (Delphi, CnS medial, Korea), which was used to minimize hemorrhage and maintain a clear visual field through the procedure. Various cutters and burrs connected to a motorized shaver (Stryker, Portage, Michigan) power console were used for soft tissue debriding and drilling.

### 2.3 Surgical procedure

The thoracolumbar BESS technique used in dogs was adapted from human BESS procedures. All dogs were premedicated with tramadol (4 mg/kg IV; Samsung Pharm, Hwasung, Korea), meloxicam (0.2 mg/kg IV; Boehringer Ingelheim, Ingelheim am Rhein, Germany), and midazolam (0.02 mg/kg IV; Bukwang Pharm, Seoul, Korea). Anesthesia was induced with propofol (6 mg/kg IV; Daewon Pharm, Seoul, Korea) and maintained with isoflurane (Hana Pharm, Seoul, Korea) at approximately 1.5 minimum alveolar concentration throughout the procedure.

Each dog was positioned in sternal recumbency and prepared for aseptic surgery. A vacuum form bag (Hug-U-Vag positioner) was placed along the lateral body wall to prevent spinal rotation. The surgeon was positioned lateral to the dog, on the side where the hemilaminectomy to be performed. The target disc space and proper alignment were confirmed using fluoroscopic guidance. Anatomic landmarks, including the dorsal spinous processes of affected vertebrae and distal tip of consecutive transverse processes, were identified and marked on the skin using a surgical skin marker.

**Figure 1:**
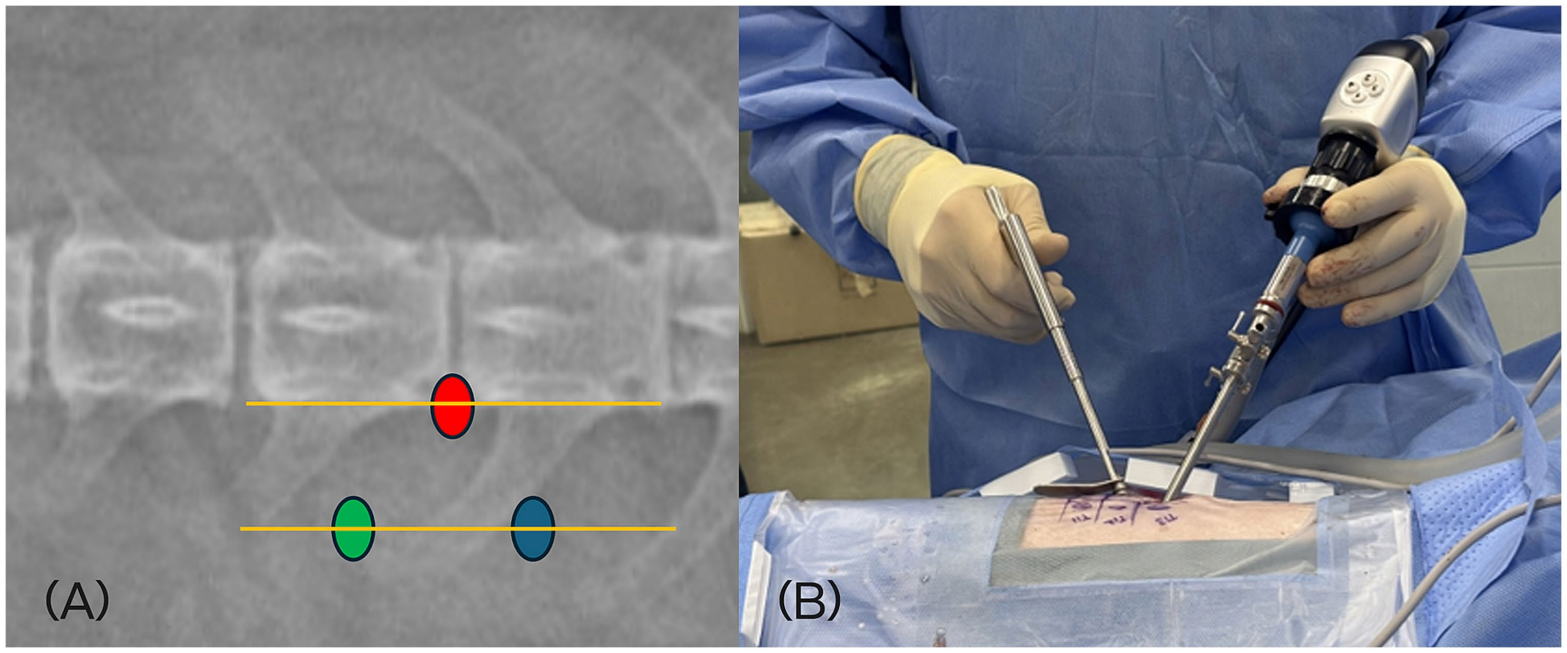
**(A)** Location of the skin incisions for left-sided L4/5 approach. The scope portal (green dot), working portal (blue dot), and docking point (red dot) at the accessory process are illustrated on a dorsoventral fluoroscopic view. **(B)** Intraoperative positioning of the surgeon. The dominant hand manipulates instruments through the working portal, while the nondominant hand controls the endoscope via the endoscopic portal.

Two portals—a working portal and an endoscopic portal—were established for the BESS approach. For left-sided lesions, the working portal was created at the level of the distal aspect of left side transverse process, approximately half a vertebral length caudal to the affected disc space and lateral to the midline. The endoscopic portal was positioned at the similarly lateral distance to the midline, approximately half a vertebral length cranial to the disc space (Figure 1). For right-sided lesions, these portal positions were mirrored. The surgeon’s dominant hand controlled instruments through the working portal, while the endoscope was handled with the nondominant hand.

**Figure 2:**
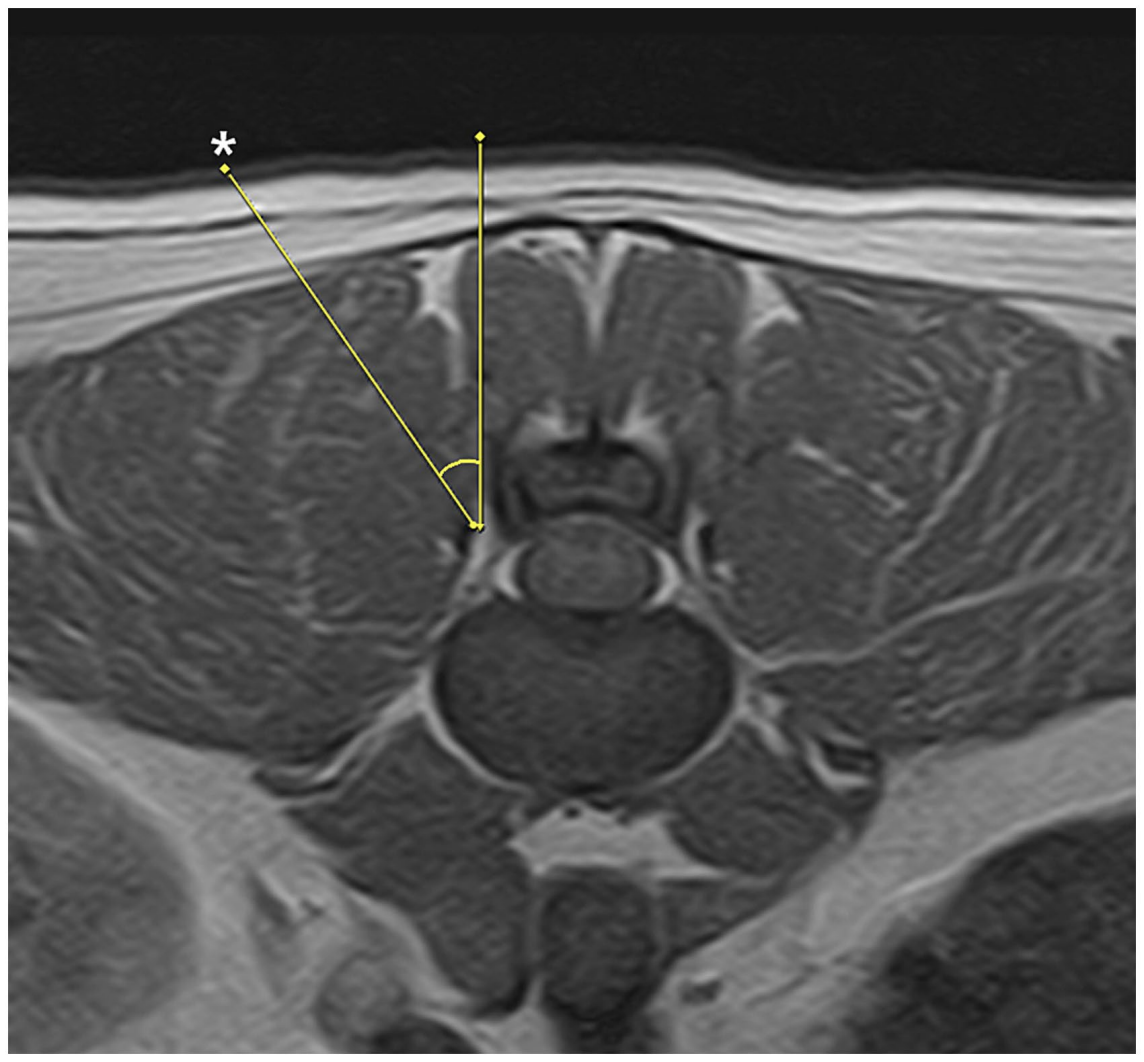
Appropriate trajectory for the paraspinal approach is 30 to 40 degrees. Skin incision points (asterisk) may vary depending on patients conformation and anatomical features.

**Figure 3:**
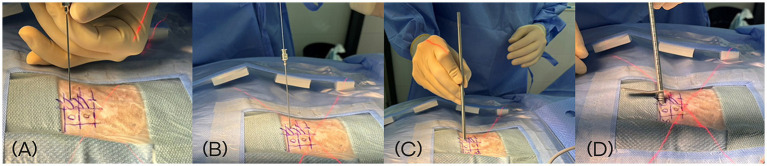
Sequential intraoperative steps for creation of the working portal during biportal endoscopic spine surgery. **(A)** Insertion of an 18 G spinal needle toward the lateral aspect of the articular process under fluoroscopic guidance. **(B)** Introduction of a 0.8 mm K-wire through the spinal needle. **(C)** Advancement of a 5-mm dilator over the K-wire following a ~ 7 mm stab incision. **(D)** Placement of the working portal cannula through the dilator after removal of the K-wire.

The docking point for the mini-hemilaminectomy was the accessory process. To optimize portal placement, preoperative MRI was reviewed to determine the appropriate distance of each incision from the midline. Skin incisions were made at an angle of approximately 30–40 degree to the skin surface (Figure 2). To create the working portal, an 18 G spinal needle was inserted through the skin lateral to the spinous process and advanced toward the lateral aspect of the articular process at the target intervertebral space under fluoroscopic guidance. Correct needle positioning was confirmed fluoroscopically. Once the docking point was verified, a 0.8 mm K-wire was inserted through the spinal needle, which was then removed. A ~ 7 mm mediolateral or craniocaudal stab incision was made at the working portal site through the skin and thoracolumbar fascia using a No. 11 scalpel blade, oriented parallel to the trajectory of the spinal needle. A 5-mm dilator was advanced over the K-wire to the lamina, followed by placement of a cannula. The K-wire and dilator were then withdrawn, leaving the cannula in place (Figure 3). A second portal, the endoscopic portal (~5 mm), was established using the same technique, except without cannula placement.

**Figure 4:**
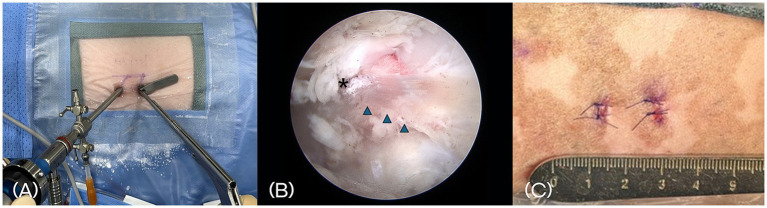
**(A)** Placement of a 4-mm, 0° rigid endoscope with an irrigation sheath into the endoscopic portal for continuous fluid irrigation. A 5-mm cannula is placed in the working portal to accommodate surgical instruments. **(B)** Intraoperative endoscopic image showing anatomical landmarks, including the accessory process (asterisk) and the longissimus lumborum muscle bundle (arrowhead). **(C)** Postoperative photograph of the skin at the surgical site. Two small stab incisions closed with nonabsorbable monofilament sutures.

A 4-mm diameter, 0-degree rigid endoscope (Stryker, Portage, Michigan) equipped with an irrigation sheath was inserted through the endoscopic portal and advanced to the lamina for visualization. This portal was used for continuous visualization of the surgical field, aided by constant irrigation with sterile saline to distend the area and clear blood or debris. The cannula placed in the working portal served to maintain a stable access channel for surgical instruments and allowed smooth saline flow (Figure 4A). Water pressure was maintained between 30 and 50 mmHg.

Paraspinal soft tissues were elevated using a periosteal elevator to expose the lamina and accessory processes. A motorized shaver and a RF probe were then used to remove residual paraspinal muscle and shrink surrounding connective tissue, thereby creating a clear working space. Key anatomical landmarks—including the accessory process and the longissimus lumborum muscle—were identified to guide the surgical approach (Figure 4B).

Once the working space was established, a mini-hemilaminectomy was performed using a high-speed drill and Kerrison rongeur to remove the laminar bone over the affected disc. After removal of the extruded disc material, the paraspinal muscle and fascia were left unsutured, and only the skin was closed using nonabsorbable sutures (Figure 4C).

### 2.4 Postoperative management

Postoperative care was similar to that provided for dogs undergoing conventional hemilaminectomy. Each dog recovered from anesthesia in the intensive care unit under standard monitoring. Postoperative analgesia was multimodal and consisted of a non-steroid anti-inflammatory drug (Meloxicam 0.2 mg/kg IV intraoperatively, followed by 0.1 mg/kg orally for 3 days up to 7 days) and an opioid (hydromorphone 0.05 mg/kg IV) given immediately at the end of surgery. Dogs were assessed daily during hospitalization for pain control and neurologic status. Recheck examinations were performed at 2, 4, and 6 weeks postoperatively, and included complete physical and neurological evaluations to monitor recovery. Neurological status and locomotor function were assessed using a Modified Frankel Scale adapted for patients (18). Owner satisfaction was evaluated subjectively through verbal questionnaires administered during recheck consultations. Any complications such as surgical site infection, worsening neurologic status, or need for additional surgery were recorded.

## 3 Results

### 3.1 Signalment and clinical signs

Thirteen dogs met the inclusion criteria. The median age was 8.5 years (range 5–15 years) and the median body weight was 5.5 kg (range 3.8–13.1 kg). The breeds represented were Dachshund (*n* = 4), Poodle (*n* = 3), Maltese (*n* = 2), French Bulldog (*n* = 2), Papillon (*n* = 1), and Bichon Frise (*n* = 1). The cohort included six neutered males, four spayed females, two intact females, and one intact male. All dogs presented with paraparesis, either ambulatory or non-ambulatory. MRI confirmed a single-level thoracolumbar disc herniation in each case. The affected disc spaces were T12–13 (*n* = 1), T13–L1 (*n* = 3), L1–2 (*n* = 2), L2–3 (*n* = 2), L3–4 (*n* = 2), L4–5 (*n* = 2), and L5–6 (*n* = 1).

### 3.2 Surgery and intraoperative complications

**Figure 5:**
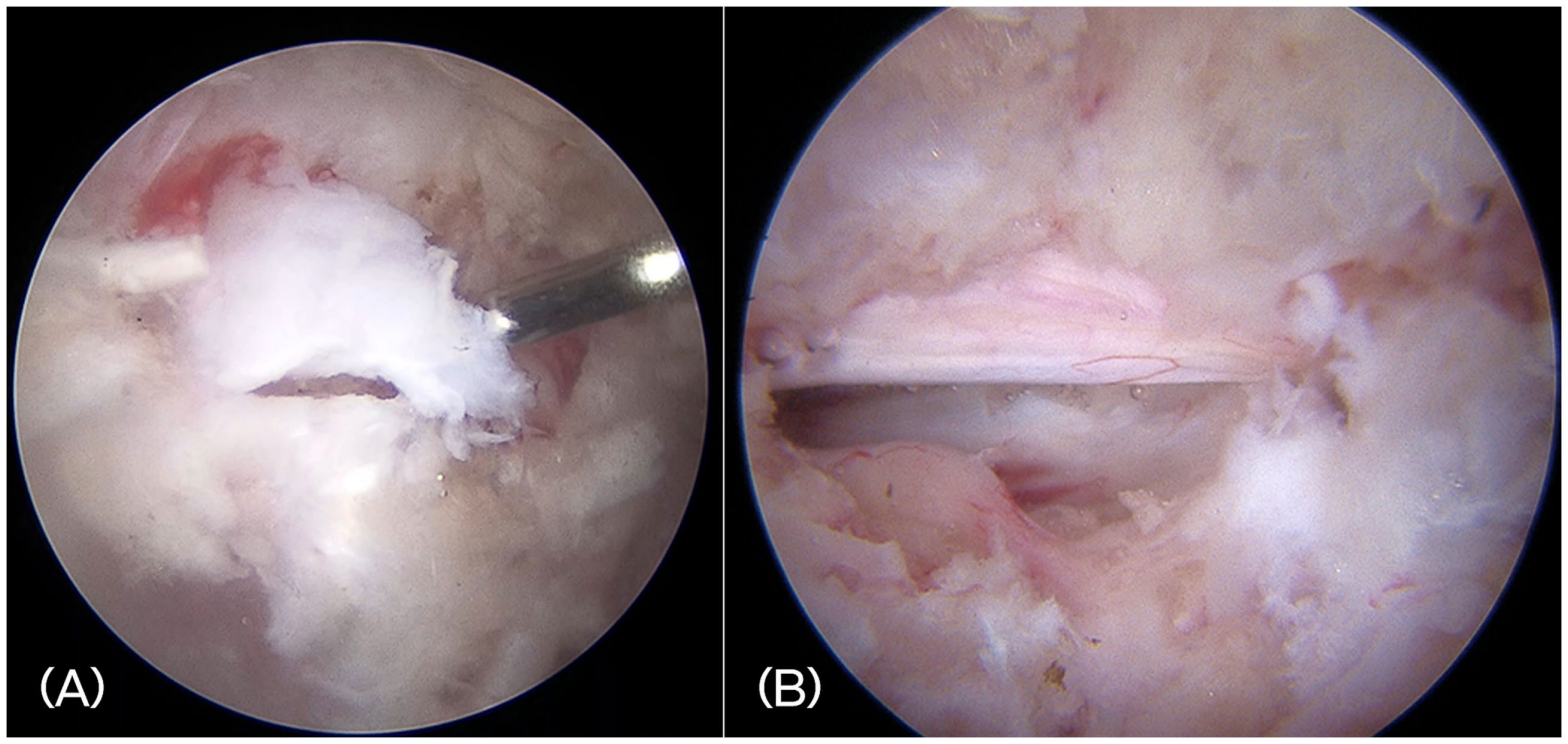
**(A)** Intraoperative endoscopic image showing the extruded disc materials. **(B)** Visualization of the decompressed spinal cord and exiting nerve root after complete removal of disc fragments.

In all cases, a mini-hemilaminectomy was performed using the BESS. Under endoscopic visualization, extruded disc material compressing the spinal cord was identified and removed. Grasping forceps and a spinal probe, inserted through the working portal, were used to extract disc fragments while minimizing retraction or manipulation of the spinal cord. The ventral aspect of the spinal cord was visualized and inspected to ensure no disc fragments remained (Figure 5). Hemostasis was achieved as needed using a small-diameter RF probe or temporarily applying hemostatic sponges.

The mean surgery time of all dogs were 53 ± 10.5 min. In three dogs, an arthroscopic power shaver was used to debride peri-foraminal soft tissue structures surrounding the bases of the transverse process of the caudal vertebrae and the accessory process. In these cases, the mean surgical time was 45 ± 4.9 min, compared to 56 ± 9.4 min in 10 dogs in which a power shaver was not used. In all dogs, major intraoperative complications did not occur.

### 3.3 Postoperative outcomes

**Figure 6:**
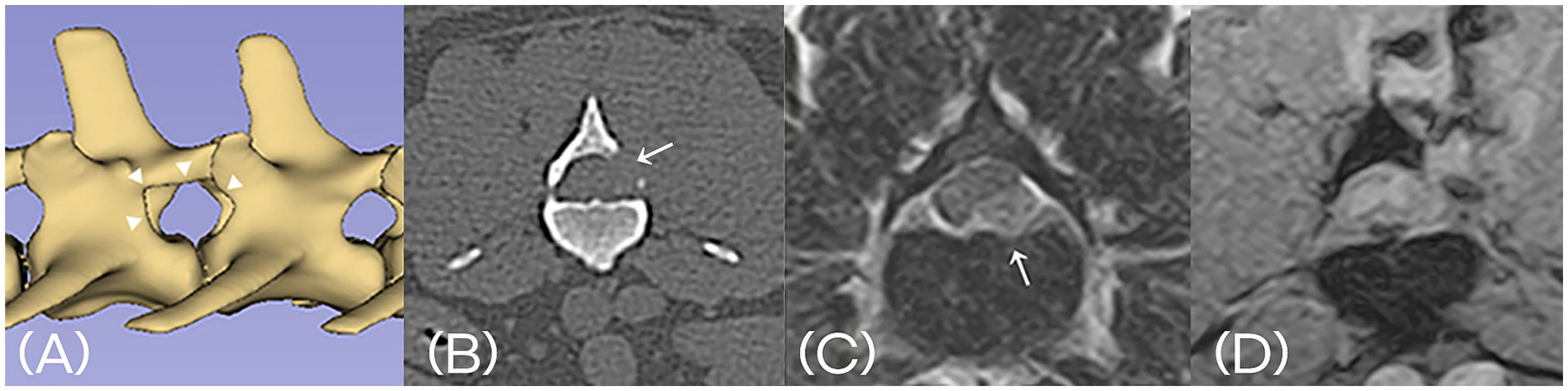
**(A)** Postoperative 3D-reconstructed CT image confirming the appropriate size and location of mini-hemilaminectomy (arrowhead). **(B)** Postoperative transverse CT image at the level of the laminectomy (arrow). **(C)** Preoperative T2-weighted MRI shows extruded intervertebral disc material compressing the spinal cord (arrow). **(D)** Postoperative T2*-weighted MRI confirms successful removal of the disc material and resolution of spinal cord compression. A fluid-filled tract is visible along the surgical access path.

Postoperative CT and MRI were performed in two cases. Postoperative CT or MRI is not routinely performed following decompression surgery. It was performed only in select cases where owner consent was obtained for clinical study purposes. CT imaging confirmed the appropriate size and location of each mini-hemilaminectomy. However, the extent of bone removal varied depending on the location and distribution of the herniated disc material. Follow-up MRI demonstrated decompression of spinal cord after removal of the extruded disc material (Figure 6).

Only mild soft tissue swelling was noted at the surgical site postoperatively, likely due to irrigation fluid accumulation; this swelling resolved within 2–3 days following the application of a soft padded bandage. No hematomas, seromas, or other wound-related complications were observed. All dogs were discharged from the hospital within 7 days of postoperatively.

The median preoperative neurological grade was grade 3 (non-ambulatory paraparesis; grade range 2–4). At 2 weeks after surgery, the median score improved to grade 2 (ambulatory paraparesis; grade range 0–4). By 6 weeks postoperatively, the median neurological grade was grade 0 (normal gait; grade range 0–2), indicating progressive and favorable neurological recovery in most patients. Gait, proprioception, and spinal reflexes were assessed as normal during clinical examination. No cases of postoperative neurologic deterioration or recurrence of clinical signs were observed. Owners reported satisfactory recovery and return to normal activity levels in all dogs.

## 4 Discussion

In this case series, we demonstrated the use of a BESS approach for thoracolumbar decompression in dogs with IVDH. Using the BESS technique, we successfully accessed the vertebral canal and achieved clear visualization of the spinal cord and nerve roots following removal of the extruded disc material. No major perioperative complications occurred, and conversion to an open hemilaminectomy was not required in any case. Clinical signs improved during the postoperative period without any deterioration. These findings suggested that BESS is a safe and feasible technique for small breed dogs and may serve as an effective surgical option for the treatment of thoracolumbar IVDH in canine patients.

BESS was initially developed in human medicine. In the early 1980s, Forst and Hausmann first introduced the arthroscope for intradiscal use (19). By the early 21st century, several authors had described endoscopic spinal decompression techniques aimed at preserving paraspinal musculature and posterior spinal stabilizing structures (20, 21). In human clinical practice, BESS has demonstrated distinct advantages over traditional open or microscopic surgery, including smaller incisions, less muscle retraction, decreased bleeding, less postoperative pain, and faster recovery (22).

In veterinary medicine, open hemilaminectomy has long been the gold-standard treatment for thoracolumbar IVDH, effectively relieving spinal cord compression by removing extruded disc material. However, this technique requires relatively extensive soft tissue dissection and bony resection, which can alter the integrity of the paraspinal muscles (23). In contrast, our application of BESS achieved the same decompressive goal using two small portals, with minimal disruption to musculature and a limited decompressive window. While this study did not include objective assessments of muscle trauma or postoperative pain, the use of small incisions, the absence of fascial closure, and the lack of wound-related complications suggest that BESS may reduce surgical trauma compared to conventional open procedures. This minimally invasive approach may result in reduced postoperative pain and faster functional recovery.

Assessment of postoperative pain following minimally invasive spine surgery in dogs remains limited. A previous study evaluating the use of a microscope assisted mini open approach in healthy dogs found no changes in mechanical sensory threshold testing 1 day postsurgery.^11^ In contrast traditional open approaches were associated with significant reduced mechanical thresholds at the surgical site 2 weeks postoperatively (24), indicating prolonged discomfort. These findings align with human studies, in which BESS has been associated with faster mobilization and recovery compared to open surgery (22). Nonetheless, future studies incorporating validated pain scoring systems, mechanical sensory threshold testing, or imaging-based assessments of muscle integrity are needed to confirm these advantages in veterinary patients.

Although no complications occurred in this study, BESS in humans has been associated with potential risks. The most commonly reported adverse events include dural tears (1.6–4.5%), transient dysesthesia (∼2.6%), epidural hematoma (0.3–1.1%), and nerve root injury (0.5–1%) (25, 26). These risks should be acknowledged, particularly during the initial learning phase.

Surgery time for decompressive procedures is not consistently reported in the veterinary literature, making direct comparison difficult. In this study, we found that a single site mini-hemilaminectomy using BESS could be performed in a mean time of 53 ± 10.5 min. Although no statistical comparison was conducted, surgical time decreased from approximately 60 min initially to around 45 min in the final three cases with the aid of an arthroscopic power shaver. This highlights the importance of technical proficiency in the successful implementation of BESS (27). Surgeons must be familiar with endoscopic anatomy, triangulation techniques between the endoscope and instruments, and intraoperative bleeding control. These skills require focused training and hands-on experience. In this series, the surgical team had extensive experience in open hemilaminectomy and arthroscopic surgery, likely contributing to the favorable outcomes observed. In less experienced settings, the risk of complications such as incomplete decompression or iatrogenic injury may be higher. Structured training, including mentorship from experienced human or veterinary endoscopic surgeons, may recommended during the initial stages of adopting this technique. Further studies investigating the learning curve for BESS in veterinary settings are warranted.

This study has several limitations. The small sample size and short follow-up period limit the generalizability of the findings. Objective measures of postoperative pain, as well as comparative data on surgical time and neurologic recovery were not included and should be addressed in future studies. Postoperative imaging was only performed in two cases, limiting our ability to assess the completeness of decompression and to detect subclinical complications such as residual disc material or epidural hematomas. Neurological assessments were performed by the surgical team without blinding, introducing potential observer bias. Additionally, pain and recovery were evaluated based on clinical observations and owner reports rather than standardized or validated scoring systems.

Other limitations included the restriction of the study population to small breed dogs with single level thoracolumbar IVDH, which may limit the applicability of the findings to larger dogs or those with more extensive or multilevel lesions. The learning curve for BESS was not formally evaluated, although surgical time appeared to improve over the course of the study. Further investigation into the training requirements and reproducibility in less experienced settings is warranted. Finally, while BESS is presumed to preserve paraspinal musculatures, this study did not include histologic or imaging-based assessment to objectively evaluate muscle integrity post operatively, nor did measure clinical benefits such as recurrence rate or prolonged discomforts in a quantifiable way.

In summary, BESS is a technically feasible and potentially effective minimally invasive technique for thoracolumbar decompression in dogs with IVDH. This approach provides excellent visualization of the spinal canal, including the ventral spinal cord, while minimizing surgical trauma. These preliminary clinical results support the safety and feasibility of BESS in dogs and suggest that it may offer a valuable alternative to conventional open surgery for treating thoracolumbar IVDH. Further studies are warranted to validate its long-term outcomes and define its role in clinical veterinary neurosurgery.

## Data Availability

The original contributions presented in the study are included in the article/supplementary material, further inquiries can be directed to the corresponding author/s.
